# Comparing psychological distress in Australians before and during the COVID-19 pandemic

**DOI:** 10.1080/00049530.2023.2207667

**Published:** 2023-05-11

**Authors:** Jack W. Klein, Garrett Tyler-Parker, Brock Bastian

**Affiliations:** a Melbourne School of Psychological Sciences, University of Melbourne, Parkville, Australia; b Research School of Management, Australian National University, Canberra, Australia

**Keywords:** Kessler psychological distress scale, K10, psychological distress, COVID-19, lockdown

## Abstract

**Objective:**

To determine if psychological distress has increased during the COVID-19 pandemic, and to identify predictors of distress.

**Method:**

Kessler Psychological Distress Scale (K10) scores from nationally representative Australian samples before (*n* = 955) and during (*n* = 1173) the pandemic were compared. The pandemic sample also completed additional COVID-19 attitudinal scales.

**Results:**

The pandemic sample reported significantly higher distress than the pre-pandemic sample, especially among Melbourne residents, women, and younger and older Australians. Stress attributed to COVID-19, feeling the pandemic management is out of control, and an unwillingness to vaccinate were also predictive of psychological distress.

**Conclusions:**

Women, youth, and Melbourne residents were most vulnerable to the negative effects of COVID-19 on wellbeing, while feelings related to a loss of control, stress about the virus, and vaccine hesitancy may have also contributed to psychological distress.

## KEY POINTS

**What is already known about this topic:**

(1) Correlational research suggests the COVID-19 pandemic has increased levels of psychological distress among Australians.
(2) This may be more pronounced among women, youth, and those that have endured extended lockdowns.
(3) Few studies have explicitly compared distress from pandemic and pre-pandemic samples.

**What this topic adds:**

(1) Comparisons between nationally representative surveys found that Australians in the pandemic sample reported a higher level of psychological distress than the pre-pandemic survey.
(2) Distress increased most among women, Melbourne residents, and young and old Australians.
(3) Other risk factors are COVID-19 related stress, vaccine hesitancy, and feeling the pandemic management is out of control.

The Coronavirus (COVID-19) has had a profound impact on Australians in terms of both its direct health implications (AIHW, 2021) and associated indirect effects, such as increased unemployment (Cole, 2021) and restriction of movement (Jose, 2021). A further indirect effect is an increase in psychological distress, stemming from both the virus itself and attempts to reduce community transmissions, such as lockdowns which have been associated with negative psychological effects (Brooks et al., 2020). While the pandemic has not increased suicides among Australians (Leske et al., 2021), despite predictions it would (Bastiampillai et al., 2020), there may have been increases in less extreme depressive and anxious symptomology. Hence, the focus of the present study is to identify whether psychological distress increased among Australians living in the pandemic compared to a pre-pandemic sample collected using the same methodology, and to identify which demographic groups were most affected.

Although it will take years to fully account for the mental health effects of the global pandemic, an impressive amount of research has already been completed. Much of the research has focused on vulnerable populations, namely medical professionals (e.g., Adams et al., 2021) and persons with existing mental or physical pathologies (e.g., Edge et al., 2021), which have reported elevated psychological distress. Research surveying general Australian pandemic samples found that almost a third of respondents reported high or very high levels of psychological distress (Rahman et al., 2020), roughly mirroring the global average (Geirdal et al., 2021). Other research has found that psychological distress was most pronounced among Australian women (Gurvich et al., 2021) and youth (Isaac et al., 2021), despite older people being most vulnerable to the virus (Mueller et al., 2020). Although suggestive, the lack of a pre-pandemic comparison in these studies makes it difficult to determine whether the pandemic increased psychological distress for these demographics, or whether these results reflect existing pre-pandemic disparities in mental health.

Several studies have also attempted to compare pandemic mental health levels to pre-pandemic norms. Rogers and Cruickshank (2021) asked participants during the pandemic to report on negative emotions felt in the past month and how they felt the same time last year. Although participants reported lower well-being compared to the previous year, research indicating the poor accuracy of attempts to recall past affect (Wilson et al., 2003) may undermine these results. Some studies have directly compared psychological distress in pandemic and pre-pandemic samples, such as correlational research which found that Australian depressive and anxiety symptomology is higher versus pre-pandemic population norms, even for those without existing mental health diagnoses (Dawel et al., 2020). Likewise, Botha et al. (2022) compared a pre-pandemic sample to a pandemic sample from July 2020 and found an increase in psychological distress. They unexpectedly did not find that the increase was greater among Victorians, despite the protracted Melbourne lockdown, and reported a greater increase in distress among Australian men versus women.

Several longitudinal studies have also been conducted. The Australian Bureau of Statistics (ABS) measured psychological distress using the Kessler Psychological Distress Scale (K10) over several months in their Household Impacts of COVID-19 Survey (ABS, 2021a) and found an initial large increase in psychological distress in August 2020, followed by an abrupt decline and stabilisation of scores from November 2020 to June 2021. Similarly, a longitudinal study by the Australian National University (ANU) Centre for Social Research and Methods tracked K6 scores (a shortened version of the K10) throughout the pandemic. K6 scores from the same respondents were reported as significantly higher at the start of the pandemic (April 2020) compared to data from several years prior (February 2017; Biddle & Gray, 2020), and, despite some fluctuations, have remained elevated until at least April 2022 (Biddle et al., 2022). Finally, Botha et al. (2023) found that psychological distress increased particularly strongly in response to longer lockdowns, although this study lacked a pre-pandemic baseline sample.

Although these studies strongly suggest psychological distress has increased, a common methodological issue is that any comparisons to pre-pandemic norms may be confounded by a change in methodology. While the pandemic has forced most surveys to be administered online, pre-pandemic psychological distress has been almost exclusively measured via face-to-face interviews or Computer-Assisted Telephone Interviewing (CATI); given there is evidence that respondents report higher psychological distress via online surveys versus face-to-face or CATI surveys (Klein et al., 2021), it is difficult to determine if an increase in K10 scores represents more psychological distress in response to the pandemic or a change in methodology. For instance, Dawel et al. (2020) compared pandemic data to foreign norms based off face-to-face surveys (e.g., Kroenke et al., 2001), while pre-pandemic ABS data has been almost exclusively collected using face-to-face interviews (e.g., Slade et al., 2011). Indeed, Botha et al. (2022) notes explicitly that they compared their data to a pre-pandemic sample collected using different methodologies and recruitment methods. Biddle and Gray (2020) used a mixture of online and CATI administration for both their pre-pandemic and pandemic surveys and may be less affected by this methodological confound, although it is not clear what proportion of the pre-pandemic sample completed the survey online and whether this was consistent between survey waves.

Moreover, it is likely that certain attitudes and beliefs exacerbated psychological distress in the pandemic. Previous research has found, unsurprisingly, that worries about COVID-19 are associated with higher psychological distress (Moore & Lucas, 2021), however less obvious attitudinal predictors may also exist. For instance, it is plausible that vaccine hesitancy, caused by concerns about the safety of the vaccine or anxiety over potential “vaccine mandates” (Kinsella & Dunstan, 2021), could play a role in increasing psychological distress. However, the literature to date is mixed, with some studies reporting a link between vaccine hesitancy and increased psychological distress or depression/anxiety (Sekizawa et al., 2022; Xu et al., 2021), and others finding no relationship (e.g., Killgore et al., 2021). Relatedly, some research has pointed to a connection between mistrust of the authorities and conspiratorial thinking, such as a belief that the government or media may be lying about the pandemic, and increased psychological distress (Chen et al., 2020; De Coninck et al., 2021). More generally, research has found a link between a perceived lack of control and psychological distress (e.g., Jiménez et al., 2017; Papanikolaou et al., 2013), suggesting that people who perceive that management of the pandemic is out of control may be more likely to exhibit increased psychological distress. Research focusing on the attitudinal predictors of psychological distress, and whether they are more or less prevalent in different demographics among the Australian population, is warranted.

The present repeated cross-sectional study seeks to compare psychological distress among Australians to a pre-pandemic sample by comparing K10 results from nationally representative surveys conducted in January 2018 (*Pre-pandemic wave*), prior to the onset of the COVID-19 pandemic, and September 2021 (*Pandemic wave*). Both surveys were collected using an online survey, allowing for a comparison between the pre-pandemic and pandemic waves without the potential confound of a change in methodologies. The study will also investigate demographic factors that have previously been associated with pandemic-related psychological distress, such as gender (Gurvich et al., 2021), age (Isaac et al., 2021), and residency in a city that has endured a prolonged lockdown (Brooks et al., 2020), to investigate their potential role as moderators of the effects of the pandemic on psychological distress. A further study aim is to examine attitudes towards COVID-19 issues, such as vaccine hesitancy and beliefs about misinformation, in order to identify specific attitudinal predictors of psychological distress among Australians living through the pandemic.

## Method

Ethics approval for this project was granted by the University of Melbourne’s Psychology Health and Applied Sciences Human Ethics Sub-Committee (Approval ID 1953782). The data for both waves was collected by the online panel provider Lightspeed Research on behalf of an Australian market research company for reasons unrelated to the current study, and later shared with this paper’s authors. As all respondents were anonymous, it was judged impractical to obtain informed consent on a post-hoc basis and the requirement for consent was hence waived. Data was collected using random sampling stratified according to nationally representative quotes on age, gender, and location. The pre-pandemic (*n* = 1,118) and pandemic (*n* = 1,414) waves had dropout rates of 13% and 17%, respectively. The pandemic wave also included a *boost sample* of Canberra residents (*n* = 173), Australia’s capital city, for reasons unrelated to the present study. Respondents were removed if they provided nonsense responses to open-ended questions, straight-lined (i.e., selecting the same response for multiple choice questions), completed the survey in under one third of the median time, or provided incomplete information, which eliminated 163 respondents from the first wave, and 241 from the second wave. The final sample was 429 males and 526 females aged 18 to 74 for the pre-pandemic wave, and 574 males and 599 females aged 14 to 64 for the pandemic wave.

All respondents completed the K10, which is used to measure non-specific psychological distress in both general population and clinical samples (Kessler et al., 2002). The scale asks participants to indicate how they have been feeling over the past 30 days in response to ten items (e.g., “worthless”, “restless or fidgety”, and “hopeless”) on a five-point scale, ranging from “none of the time” to “all of the time”. K10 scores range from 10 to 50, with a higher score indicating greater psychological distress, and was highly reliable in both the pre-pandemic (α = .94) and pandemic wave (α = .95).

The pandemic wave respondents answered several novel additional questions aimed at assessing their attitudes towards the pandemic, and general life satisfaction. Respondents were presented with a list of 38 societal problems (e.g., “global warming”, “bushfires”, ‘the Australian economy”, “job security”, “crime”, and “COVID-19”) and asked to indicate whether each item currently concerns them, and which was their greatest concern. Participants also answered questions specifically related to the pandemic on a 5-point scale, including whether they believed Australia had COVID-19 under control, if the Australian government was doing enough to stop its spread, whether the media and state/territory government had spread misinformation about COVID-19, whether they were willing to be vaccinated against COVID-19, and whether they had experienced more stress in their life due to the COVID-19 pandemic. Finally, respondents reported whether they felt life in Australia was getting better on a 5-point scale, ranging from “a lot worse” to “a lot better”, and how happy they were with their life on a 7-point scale, ranging from “extremely happy” to “extremely unhappy”.

All respondents provided demographic information (e.g., age, gender, and region they live in), and answered questions unrelated to the present study. These questions were largely concerned with the participant’s opinions on topics related to market research (“What are the most important qualities you believe your brand should have?”) or the environment (“How knowledgeable would you say you are about the concept of sustainability?). The mean completion time was 29 minutes 29 seconds for the pre-pandemic wave, and 38 minutes 22 seconds for the pandemic wave.

## Results

Data was weighted according to age, gender, and state population estimates to be nationally representative. Weights for the pre-pandemic wave were based on ABS population estimates from Australian Demographics Statistics, June 2018 (ABS, 2018), and weights for the pandemic wave came from Australian Demographic Statistics, March 2021 (ABS, 2021b). Due to minor sampling differences between the waves (e.g., differing age ranges, a boost sample of Canberrans in pandemic wave), two weights were constructed. *Weight one* included only an age range shared between the samples (i.e., 18–64) and did not include participants in the Canberra boost sample, thereby ensuring that the samples were compared on an even basis. This weight excluded 392 participants from the pandemic wave and ranged from 0.44 to 2.42. *Weight two* weighted the pandemic wave according to its full age range (i.e., 14–64) and included participants from the Canberra boost sample. Weights ranged from 0.06 to 2.34. The smallest weight values were all associated with respondents from the Canberra boost, which comprised 15.4% (*n* = 181) of the final sample but only 1.68% of the Australian population. Excluding these respondents, the smallest weight value was .56. Weight one was used for comparisons between the pre-pandemic and pandemic waves, while weight two was used for analyses on the pandemic wave only.

Table 1 presents a comparison of mean psychological distress by age, gender, and Melbourne residency from the pre-pandemic and pandemic waves, weighted using weight one. The 95% CIs for these means were [21.32, 22.61], and [22.59, 23.88], respectively. Q-Q plots and histograms both indicated that psychological distress was slightly positively skewed in both waves, particularly the pandemic wave one (skewness = .71), although the large sample size of both waves means that minor deviations from normality should not affect parametric analyses (Ghasemi & Zahediasl, 2012). Minor non-normality and heteroskedasticity was observed in the regression models predicting psychological distress discussed below; analyses were repeated with a bootstrapping correction, but this produced very similar results and hence was not reported.

**Table 1.** Weighted K10 mean (SD) for pre-pandemic and pandemic waves, by age, gender, and Melbourne residency.

	18–24	25–34	35–44	45–54	55–64	Male	Female	Melbourne Resident	Total
Pre-pandemic wave	23.49 (7.30)	25.65 (9.13)	22.05 (8.94)	20.38 (9.88)	17.6 (7.31)	21.78 (9.56)	22.14 (8.64)	21.38 (9.66)	21.96 (9.10)
Pandemic wave	28.58 (8.62)	25.41 (9.28)	22.84 (9.25)	20.12 (9.89)	20.15 (10.09)	22.17 (9.14)	24.26 (10.53)	24.99 (10.51)	23.24 (9.92)

An independent samples t-test was conducted to compare psychological distress between the two waves. Respondents in the pandemic wave had significantly higher psychological distress than those in the pre-pandemic wave, *t*(1,601) = −2.79, *p* = .005, with a mean difference of −1.27, 95% CI [−2.17, −0.38] and small effect size *d* = −.13. Table 2 compares the proportion of respondents reporting high or very high levels of psychological distress between both waves. This was defined as any respondent with a K10 score over 22, mirroring the ABS criterion. A logistic regression found that wave was a significant predictor of high or very high levels of psychological distress χ2 (1, *N* = 1,736) = 7.43, *p* < .006. Odds ratios indicated that respondents in the pandemic wave were 1.30 times more likely to be in the high/very high distress category than respondents in the pre-pandemic wave.

**Table 2.** Percentage (SD) of sample reporting high or very high levels of psychological distress in pre-pandemic and pandemic waves, by age, gender, and Melbourne residency.

	18–24	25–34	35–44	45–54	55–64	Male	Female	Melbourne Resident	Total
Pre-pandemic wave	58.1 (0.5)	67.62 (0.47)	58.18 (0.49)	34.95 (0.48)	23.31 (0.42)	44.65 (0.5)	46.68 (0.5)	42.67 (0.5)	45.67 (0.5)
Pandemic wave	78.59 (0.41)	62.57 (0.49)	53 (0.5)	33.86 (0.47)	37.18 (0.48)	47.72 (0.5)	56.53 (0.5)	59.37 (0.49)	52.23 (0.5)

Further analyses were conducted to identify moderators of the relationship between wave and psychological distress. Age and gender were identified as potential moderators, with previous research identifying women (Gurvich et al., 2021) and youth (Isaac et al., 2021) as especially at risk of increased psychological distress. Similarly, residency of Melbourne (*N* = 346) and Sydney (*N* = 354) was used to operationalise the effects of long-term lockdowns on psychological distress. Residency in these cities were chosen as proxies for lockdowns due to their relatively large sample sizes and the prevalence of lockdowns in these regions, with Melbourne representing arguably the longest locked-down city in the world (Campbell, 2021), and Sydney the second most locked-down city in Australia (Mao, 2021). Prior to analyses age was centred, and gender and Melbourne/Sydney residency were dummy coded with “males” and “non-Melbourne/Sydney resident” used as reference categories for each variable respectively. Wave, gender, age, Melbourne/Sydney residency, and interaction terms between the moderators and wave were entered into a multiple regression model as predictors of psychological distress. Additionally, as a visual inspection of the data indicated the presence of a possible non-linear interaction between wave and age, a quadratic wave-age interaction term was included in the model.

The regression model was significant, R^2^ = .10, *Wald F* (10, 1,592) = 19.86, *p* < .000, with women (*B* = 2.24, *p* = .014) and Melbourne residents (*B* = 2.64, *p* = .034), but not Sydney residents (*B* = −1.14, *p* = .316), reporting significantly higher psychological distress in the pandemic versus pre-pandemic wave. Moreover, the quadratic (*B* = 0.01, *p* = .003), but not linear (*B* = −0.03, *p* = .450), interaction for wave with age was significant, with both younger and older Australians reporting significantly higher psychological distress in the pandemic wave. These results indicate that psychological distress rose particularly among women, Melbourne residents, and young and old Australians. Replicating Botha et al. (2022), these analyses were repeated with dummy variables for Victoria and NSW instead of Melbourne and Sydney respectively. Neither the moderation effects for Victoria (*B* = −0.48, *p* = .645) or NSW (*B* = 1.54, *p* = .188) were significant, suggesting that it was Melbourne residency specifically that predicted the increase in psychological distress.

Age, gender, and Melbourne/Sydney residency in the pandemic wave only were regressed onto psychological distress, R^2^ = .13, *Wald F* (4, 1,028) = 39.95, *p* < .000, with youth (*B* = −0.23, *p* < .000), being a woman (*B* = 2.23, *p* = .000), and Melbourne residency (*B* = 1.84, *p* = .028), but not Sydney residency (*B* = −0.74, *p* = .316), significantly predicting higher psychological distress. This suggests that youth, women, and Melbourne residents experienced the highest levels of psychological distress during the COVID-19 pandemic in Australia.

83.1% of the pandemic wave was currently concerned by COVID-19, and 24.2% indicated that COVID-19 (22.1%) or a disease outbreak/epidemic (2%) was their greatest concern, surpassing the cost of living/affordable housing (17.2%) and climate change/global warming (15.5%). To test the impact of COVID-19 beliefs on psychological distress, several items assessing attitudes towards COVID-19 were entered into a regression model to predict psychological distress (see Table 3). The regression model was significant, R^2^ = .09, *Wald F* (6, 1,026) = 13.37, *p* < .000, with significant main effects for a belief that Australia is out of control when it comes to pandemic management, an unwillingness to get vaccinated, and an endorsement of increased stress due to the COVID-19 pandemic.

**Table 3.** COVID-19 related beliefs regressed onto psychological distress (K10).

Independent Variables	B	SE B	95% CI	*t*	*p*-value
*I feel Australia is out of control when it comes to our management of COVID-19*	0.67	0.32	[0.04,1.20]	2.07	.039
*I am not willing to get vaccinated against COVID-19*	1.05	0.26	[0.54,1.55]	4.04	<.001
*The media spreads misinformation about COVID-19 pandemic*	0.11	0.30	[−0.45,0.69]	0.36	.716
*The Australian government could do a lot more to stop the spread of COVID-19*	0.34	0.29	[−0.24,9.2]	1.15	.250
*My state/territory government over-exaggerates their reporting of the COVID-19 pandemic*	0.08	0.31	[−0.54,0.69]	0.24	.809
*I have experienced more stress in my life due to the COVID-19 pandemic*	1.51	0.29	[0.93,2.08]	5.17	<.001

Follow-up analyses indicated that the effect of COVID-19 related stress on psychological distress was moderated by age (*B* = 0.06, *p* < .000), such that it was a stronger positive predictor of the K10 among older Australians. There was no significant interaction between stress and Melbourne residency (*B* = 0.55, *p* = .839) or gender (*B* = 0.72, *p* = .155). By contrast, the effect of a belief that Australia’s pandemic management is out of control on psychological distress was moderated by gender (*B* = 1.12, *p* = .034), but not age (*B* = 0.02, *p* = .362) or Melbourne residency (*B* = 2.78, *p* = .246), such that it was a stronger predictor of psychological distress among women. The effect of vaccine hesitancy on psychological distress was not significantly moderated by age (*B* = −0.01, *p* = .667), gender (*B* = 0.24, *p* = .576), or Melbourne residency (*B* = −0.99, *p* = .110).

Causes of psychological distress that weren’t directly related to COVID-19 were also regressed onto psychological distress in models containing age and gender, which were examined as potential moderators (see Table 4). The model containing life satisfaction was significant, R^2^ = .35, *Wald F* (5, 1,020) = 114.42, *p* < .000. This revealed a significant main effect and interactions with age and gender, such that life satisfaction was a stronger negative predictor of psychological distress among women and older Australians. Similarly, a model containing perceptions that life in Australia was getting better was significant, R^2^ = .15, *Wald F* (5, 1,027) = 40.38, *p* < .000. This again revealed a significant main effect and interactions with age, but not gender, such that a perception that life in Australia was getting better was a stronger negative predictor of psychological distress among older Australians.

**Table 4.** Life satisfaction and perception that life in Australia is getting better regressed onto psychological distress (K10), controlling for age and gender.

Model	Independent Variables	B	SE B	95% CI	*t*	*p*-value
1	Life satisfaction	−2.58	0.26	[−3.09,-2.08]	−10.03	<.001
	Age	−0.06	0.06	[−0.18,0.06]	−0.98	.328
	Gender (Female)	5.33	1.86	[1.69,8.98]	2.87	.004
	Age*Life satisfaction	−0.04	0.01	[−0.06,-0.01]	−2.77	.006
	Gender*Life satisfaction	−0.84	0.39	[−1.59,-0.08]	−2.17	.030
2	Life better	−1.04	0.39	[−1.81,-0.28]	−2.68	.008
	Age	−0.10	0.06	[−0.22,0.02]	−1.72	.086
	Gender (Female)	3.16	1.74	[−0.25,6.57]	1.82	.069
	Age*Life better	−0.06	0.02	[−0.10,-0.02]	−2.74	.006
	Gender*Life better	−0.40	0.58	[−1.53,.73]	−0.70	.487

## Discussion

The present study aimed to compare psychological distress between nationally representative pandemic and pre-pandemic samples, and to identify demographic variables that moderated the psychological effect of the pandemic. Results indicated that psychological distress had increased compared to a pre-pandemic sample, mirroring other research (Biddle & Gray, 2021). Our interaction analyses, which examined age, gender, and Melbourne residency as moderators, supports previous research that identified Australian youth (Isaac et al., 2021), those that have experienced an extended lockdown (Botha et al., 2023; Brooks et al., 2020), and women (Gurvich et al., 2021), as particularly vulnerable to the psychological effects of the pandemic. This also contrasts the somewhat unexpected findings of Botha et al. (2022) which reported a relatively greater elevation in distress for men. Interestingly, although we found that psychological distress increased most for younger and older Australians, distress for older Australians overall was substantially lower than that reported by younger Australians, suggesting that younger people were at most risk. Moreover, while Melbourne residency significantly predicted psychological distress, Sydney residency did not; considering that Melbourne had longer and harsher lockdowns than Sydney (Manheim, 2021), this may suggest that only particularly prolonged lockdowns have a substantial effect on psychological distress. Like Botha et al. (2022), we did not find an effect of Victorian residency on psychological distress. This is likely because the lockdowns in rural Victoria were far shorter and less restrictive than those in Melbourne, further suggesting that only particularly extreme lockdown measures take a toll on wellbeing.

A further aim of the study was to examine how attitudes towards COVID-19 were associated with increased psychological distress. Most Australians were concerned with COVID-19, representing the top concern of roughly one fifth of respondents. Indeed COVID-19 related stress was a positive predictor of psychological distress, particularly among older Australians, which is relatively unsurprising given that COVID-19 poses a greater mortality risk to older Australians (Mueller et al., 2020). Psychological distress was also related to a perception that the pandemic was out of control, particularly among women, reflecting previous research that has linked perceived lack of control and psychological distress (e.g., Jiménez et al., 2017). The positive relationship between unwillingness to be vaccinated and psychological distress suggests that vaccine hesitancy may increase psychological distress, mirroring other research (e.g., Xu et al., 2021). Moreover, these results point to the psychologically damaging effects of a pandemic misinformation environment so extreme that is has been labelled by the World Health Organisation as an *infodemic* (Skafle et al., 2022), and suggest that vaccine misrepresentations may have taken a tangible toll on mental health. Nevertheless, the model using COVID-19 attitudes to predict psychological distress only predicted 9% of the variance in psychological distress, suggesting that beliefs about the pandemic may only be responsible for a small portion of the increase in psychological distress. A more likely explanation is these items only captured COVID-19 related stress in a narrow sense, in terms of the virus itself, rather than its downstream consequences (e.g., lockdowns, unemployment) which also contributed to diminished mental health.

It is worth considering whether psychological distress levels will return to pre-pandemic levels as concerns about COVID-19 eventually dissipate. Longitudinal data suggests that psychological distress levels in Australia remained elevated until at least April 2022 (Biddle et al., 2022). If the increase in psychological distress among older Australians is related to stress directly attributed to COVID-19, as the present study’s results suggest, then it should reduce as the virus becomes a less salient threat. It is less clear whether the same will be true for younger Australians for whom psychological distress was less tied to stress over COVID-19, or even general life satisfaction. There has been a steady increase in youth psychological distress that pre-dates the COVID-19 pandemic (Brennan et al., 2021), which may be underwritten by stressors such as the widespread use of social media among youth (Lin et al., 2016). There is also evidence that youth were particularly vulnerable to stress during the pandemic (Varma et al., 2021), with pandemic disruptions, such as the closure of universities and increased social isolation, perhaps exacerbating an existing trend. It is unclear to what degree youth psychological distress can be directly attributed to the current pandemic, or if it will decrease as COVID-19 concerns recede.

This research has several strengths. Most importantly, it compared psychological distress between samples that used a consistent online survey methodology. This eliminated the potential confound of changing methodologies influencing K10 scores, which may have occurred in other comparative studies (e.g., Dawel et al., 2020). The present study was also collected and weighted according to nationally representative demographic quotas, allowing for conclusions that may more accurately represent the Australian population than studies which have over-sampled a particular gender (e.g., Rogers & Cruickshank, 2021), age (e.g., Geirdal et al., 2021), or residents of a particular state (e.g., Rahman et al., 2020). A potential criticism is that our sample was composed of panel respondents, which may differ in psychological distress from the general population. However, as the primary research purpose was to examine changes in psychological distress rather than necessarily provide an accurate estimate of mean psychological distress in Australia, our primary concern was ensuring that the waves were comparable. Hence, unless panel respondents were differentially affected by the pandemic versus the general population, any potential sample selection bias is of limited concern.

There are several implications of this research. Firstly, it provides converging evidence that there was an increase in psychological distress among Australians due to the pandemic, suggesting that COVID-19 and the Government strategies to contain it have had a tangible effect on mental health. Our findings also identified Australian youth, women, and residents of Melbourne as having been particularly vulnerable to the negative mental health effects of the pandemic, as were people with COVID-19 related anxieties, such as an unwillingness to vaccinate or concern over how the pandemic is being handled. The latter finding raises concerns about the culture of misinformation that permeated the pandemic, particularly related to vaccine hesitancy, and the effects it may have had on wellbeing. Future research should continue to monitor psychological distress as COVID-19 concerns subside and examine if mental health outcomes subsequently improve. This is particularly important for demographics with psychological distress elevation less clearly linked to the virus itself, such as younger Australians.

## Conclusion

The present paper has found that psychological distress among a nationally representative pandemic online sample has increased relative to a pre-pandemic sample. This was particularly true for youth, females, and residents of Melbourne, which appeared to be the demographic groups most at risk of increased psychological distress. Concerns that the COVID-19 management is out of control and an unwillingness to vaccinate were also identified as risk factors, with stress related to the virus itself particularly predictive of psychological distress among older Australians. Future research should continue to monitor psychological distress and examine whether psychological distress return to their pre-pandemic levels as concerns about COVID-19 eventually fade, particularly among Australian youth.

## Data Availability

The data that support the findings of this study are openly available in OSF at http://doi.org/10.17605/OSF.IO/YRN49, reference number [yrn49].

## Open Scholarship

This article has earned the Center for Open Science badge for Open Data. The data are openly accessible at https://osf.io/j3hga/?view_only=d676f0cba3634f86bc45ccb215b95bd3

## References

[cit0001] ABS. (2018, June). Australian demographic statistics. https://www.abs.gov.au/AUSSTATS/abs@.nsf/DetailsPage/3101.0Jun%202018?OpenDocument

[cit0002] ABS. (2021a). Household impacts of COVID-19 survey. https://www.abs.gov.au/statistics/people/people-and-communities/household-impacts-covid-19-survey/latest-release

[cit0003] ABS. (2021b). *National, state and territory population*. Retrieved March, 2021, form https://www.abs.gov.au/statistics/people/population/national-state-and-territory-population/latest-release

[cit0004] Adams, M. A., Brazel, M., Thomson, R., & Lake, H. (2021). The mental health of Australian medical practitioners during Covid-19. *Australasian Psychiatry*, 29(5), 523–9. 10.1177/10398562211010807 34010578

[cit0005] AIHW. (2021). *The first year of COVID-19 in Australia: Direct and indirect health effects*.

[cit0006] Bastiampillai, T., Allison, S., Looi, J. C. L., Licinio, J., Wong, M. L., & Perry, S. W. (2020). The COVID-19 pandemic and epidemiologic insights from recession-related suicide mortality. *Molecular Psychiatry*, 25(12), 3445–3447. 10.1038/s41380-020-00875-4 32873897 PMC7462110

[cit0007] Biddle, N., & Gray, M. (2020). *Hardship, distress, and resilience: The initial impacts of COVID-19 in Australia*. ANU. https://csrm.cass.anu.edu.au/news/hardship-distress-and-resilience-initial-impacts-covid-19-australia

[cit0008] Biddle, N., & Gray, M. (2021). *Tracking wellbeing outcomes during the COVID-19 pandemic (October 2021): Putting the worst behind us?* ANU. https://csrm.cass.anu.edu.au/research/publications/tracking-wellbeing-outcomes-during-covid-19-pandemic-october-2021-putting

[cit0009] Biddle, N., Gray, D., & Rehill, P. (2022). *Mental health and wellbeing during the COVID-19 period in Australia*. ANU.

[cit0010] Botha, F., Butterworth, P., & Wilkins, R. (2022). Evaluating how mental health changed in Australia through the COVID-19 pandemic: Findings from the ‘Taking the Pulse of the Nation’ (TTPN) survey. *International Journal of Environmental Research and Public Health*, 19(1), 558. 10.3390/ijerph19010558 35010819 PMC8744652

[cit0011] Botha, F., Morris, R. W., Butterworth, P., & Glozier, N. (2023). Trajectories of psychological distress over multiple COVID-19 lockdowns in Australia. *SSM Popul Health*, 21, 101315. 10.1016/j.ssmph.2022.101315 36530365 PMC9742066

[cit0012] Brennan, N., Beames, J. R., Kos, A., Reily, N., Connell, C., Hall, S., Yip, D., Hudson, J., O’Dea, B., DiNicola, K., & Christie, R. (2021). Psychological distress in young people in Australia-Fifth annual biennial youth mental health report: 2012-2020. Mission Australia.

[cit0013] Brooks, S. K., Webster, R. K., Smith, L. E., Woodland, L., Wessely, S., Greenberg, N., & Rubin, G. J. (2020). The psychological impact of quarantine and how to reduce it: Rapid review of the evidence. *The Lancet*, 395(10227), 912–920. 10.1016/s0140-6736(20)30460-8 PMC7158942 32112714

[cit0014] Campbell, D. (2021, October 25). Josh Frydenberg says Melbourne is the world’s most locked down city. Is that correct? *ABC News*. https://www.abc.net.au/news/2021-10-25/fact-check-is-melbourne-most-locked-down-city/100560172

[cit0015] Chen, X., Zhang, S. X., Jahanshahi, A. A., Alvarez-Risco, A., Dai, H., Li, J., & Ibarra, V. G. (2020). Belief in a COVID-19 conspiracy theory as a predictor of mental health and well-being of health care workers in Ecuador: Cross-sectional survey study. *JMIR Public Health and Surveillance*, 6(3), e20737. 10.2196/20737 32658859 PMC7375774

[cit0016] Cole, W. (2021, September 16). Australian employment slides in August as lockdowns slash workers’ hours. *Reuters*. https://www.reuters.com/world/asia-pacific/australian-employment-falls-by-146000-jobs-aug-lockdowns-hit-2021-09-16/

[cit0017] Dawel, A., Shou, Y., Smithson, M., Cherbuin, N., Banfield, M., Calear, A. L., Farrer, L. M., Gray, D., Gulliver, A., Housen, T., McCallum, S. M., Morse, A. R., Murray, K., Newman, E., Rodney Harris, R. M., & Batterham, P. J. (2020). The effect of COVID-19 on mental health and wellbeing in a representative sample of Australian adults. *Frontiers in Psychiatry*, 11, 579985. 10.3389/fpsyt.2020.579985 33132940 PMC7573356

[cit0018] De Coninck, D., Frissen, T., Matthijs, K., d’Haenens, L., Lits, G., Champagne-Poirier, O., Carignan, M. E., David, M. D., Pignard-Cheynel, N., Salerno, S., & Genereux, M. (2021). Beliefs in conspiracy theories and misinformation about COVID-19: Comparative perspectives on the role of anxiety, depression and exposure to and trust in information sources. *Frontiers in Psychology*, 12, 646394. 10.3389/fpsyg.2021.646394 33935904 PMC8085263

[cit0019] Edge, R., Mazariego, C., Li, Z., Canfell, K., Miller, A., Koczwara, B., Shaw, J., & Taylor, N. (2021). Psychosocial impact of COVID-19 on cancer patients, survivors, and carers in Australia: A real-time assessment of cancer support services. *Supportive Care in Cancer: Official Journal of the Multinational Association of Supportive Care in Cancer*, 29(9), 5463–5473. 10.1007/s00520-021-06101-3 33694089 PMC7946616

[cit0020] Geirdal, A. O., Ruffolo, M., Leung, J., Thygesen, H., Price, D., Bonsaksen, T., & Schoultz, M. (2021). Mental health, quality of life, wellbeing, loneliness and use of social media in a time of social distancing during the COVID-19 outbreak. A cross-country comparative study. *Journal of Mental Health*, 30(2), 148–155. 10.1080/09638237.2021.1875413 33689546

[cit0021] Ghasemi, A., & Zahediasl, S. (2012). Normality tests for statistical analysis: A guide for non-statisticians. *International Journal of Endocrinology and Metabolism*, 10(2), 486–489. 10.5812/ijem.3505 23843808 PMC3693611

[cit0022] Gurvich, C., Thomas, N., Thomas, E. H., Hudaib, A. R., Sood, L., Fabiatos, K., Sutton, K., Isaacs, A., Arunogiri, S., Sharp, G., & Kulkarni, J. (2021). Coping styles and mental health in response to societal changes during the COVID-19 pandemic. *The International Journal of Social Psychiatry*, 67(5), 540–549. 10.1177/0020764020961790 33016171

[cit0023] Isaac, V., Cheng, T., Townsin, L., Assareh, H., Li, A., & McLachlan, C. S. (2021). Associations of the initial COVID-19 lockdown on self-reported happiness and worry about developing loneliness: A cross-sectional analysis of rural, regional, and urban Australian communities. *International Journal of Environmental Research and Public Health*, 18(18). 10.3390/ijerph18189501 PMC8467504 34574425

[cit0024] Jiménez, M. G., Montorio, I., & Izal, M. (2017). The association of age, sense of control, optimism, and self-esteem with emotional distress. *Developmental Psychology*, 53(7), 1398–1403. 10.1037/dev0000341 28459257

[cit0025] Jose, R. (2021, 21 October). Melbourne readies to exit world’s longest COVID-19 lockdown. *Reuters*. https://www.reuters.com/world/asia-pacific/melbourne-readies-exit-worlds-longest-covid-19-lockdowns-2021-10-20/

[cit0026] Kessler, R. C., Andrews, G., Colpe, L. J., Hiripi, E., Mroczek, D. K., Normand, S. L. T., Walters, E. E., & Zaslavsky, A. M. (2002). Short screening scales to monitor population prevalences and trends in non-specific psychological distress. *Psychological Medicine*, 32(6), 959–976. 10.1017/s0033291702006074 12214795

[cit0027] Killgore, W. D. S., Cloonan, S. A., Taylor, E. C., & Dailey, N. S. (2021). The COVID-19 vaccine is here—Now who is willing to get it? *Vaccines (Basel)*, 9(4), 339. 10.3390/vaccines9040339 33916161 PMC8065766

[cit0028] Kinsella, E., & Dunstan, J. (2021, Nov 17). Debate around Victoria’s COVID-19 pandemic bill has turned ugly. Here’s what you need to know. *ABC News*. https://www.abc.net.au/news/2021-11-17/victoria-covid-pandemic-bill-daniel-andrews-parliament/100623972

[cit0029] Klein, J. W., Tyler‐parker, G., & Bastian, B. (2021). Measuring psychological distress among Australians using an online survey. *Australian Journal of Psychology*, 72(3), 276–282. 10.1111/ajpy.12283 PMC12175680 40666009

[cit0030] Kroenke, K., Spitzer, R. L., & Williams, J. B. W. (2001). The PHQ-9: Validity of a brief depression severity measure. *Journal of General Internal Medicine*, 16(9), 606–613. 10.1046/j.1525-1497.2001.016009606.x 11556941 PMC1495268

[cit0031] Leske, S., Kõlves, K., Crompton, D., Arensman, E., & de Leo, D. (2021). Real-time suicide mortality data from police reports in Queensland, Australia, during the COVID-19 pandemic: An interrupted time-series analysis. *The Lancet Psychiatry*, 8(1), 58–63. 10.1016/s2215-0366(20)30435-1 33212023 PMC7836943

[cit0032] Lin, L. Y., Sidani, J. E., Shensa, A., Radovic, A., Miller, E., Colditz, J. B., Hoffman, B. L., Giles, L. M., & Primack, B. A. (2016). Association between social media use and depression among U.S. young adults. *Depression and Anxiety*, 33(4), 323–331. 10.1002/da.22466 26783723 PMC4853817

[cit0033] Manheim, M. (2021). A tale of three cities: How COVID-19 lockdowns restricted movement in Canberra, Sydney and Melbourne. *ABC News*. https://www.abc.net.au/news/2021-09-02/how-covid-lockdown-restricted-movement-melbourne-sydney-canberra/100427148

[cit0034] Mao, F. (2021, October 11). Covid Australia: Sydney celebrates end of 107-day lockdown. *BBC*. https://www.bbc.com/news/world-australia-58866464

[cit0035] Moore, K. A., & Lucas, J. J. (2021). COVID-19 distress and worries: The role of attitudes, social support, and positive coping during social isolation. *Psychology and Psychotherapy*, 94(2), 365–370. 10.1111/papt.12308 32981116 PMC7537287

[cit0036] Mueller, A. L., McNamara, M. S., & Sinclair, D. A. (2020). Why does COVID-19 disproportionately affect older people? *Aging*, 12(10), 9959–9981. 10.18632/aging.103344 32470948 PMC7288963

[cit0037] Papanikolaou, V., Gadallah, M., Leon, G. R., Massou, E., Prodromitis, G., Skembris, A., & Levett, J. (2013). Relationship of locus of control, psychological distress, and trauma exposure in groups impacted by intense political conflict in Egypt. *Prehospital and Disaster Medicine*, 28(5), 423–427. 10.1017/S1049023X13008601 23719509

[cit0038] Rahman, M. A., Hoque, N., Alif, S. M., Salehin, M., Islam, S. M. S., Banik, B., Sharif, A., Nazim, N. B., Sultana, F., & Cross, W. (2020). Factors associated with psychological distress, fear and coping strategies during the COVID-19 pandemic in Australia. *Globalization and Health*, 16(1), 95. 10.1186/s12992-020-00624-w 33032629 PMC7542573

[cit0039] Rogers, S. L., & Cruickshank, T. (2021). Change in mental health, physical health, and social relationships during highly restrictive lockdown in the COVID-19 pandemic: Evidence from Australia. *PeerJ*, 9, e11767. 10.7717/peerj.11767 34327055 PMC8310621

[cit0040] Sekizawa, Y., Hashimoto, S., Denda, K., Ochi, S., & So, M. (2022). Association between COVID-19 vaccine hesitancy and generalized trust, depression, generalized anxiety, and fear of COVID-19. *BMC Public Health*, 22(1), 126. 10.1186/s12889-021-12479-w 35042506 PMC8764499

[cit0041] Skafle, I., Nordahl-Hansen, A., Quintana, D. S., Wynn, R., & Gabarron, E. (2022). Misinformation about COVID-19 vaccines on social media: Rapid review. *Journal of Medical Internet Research*, 24(8), e37367. 10.2196/37367 35816685 PMC9359307

[cit0042] Slade, T., Grove, R., & Burgess, P. (2011). Kessler psychological distress scale: Normative data from the 2007 AustraliaN national survey of mental health and wellbeing. *The Australian and New Zealand Journal of Psychiatry*, 45, 308–316. 10.3109/00048674.2010.543653 21332432

[cit0043] Varma, P., Junge, M., Meaklim, H., & Jackson, M. L. (2021). Younger people are more vulnerable to stress, anxiety and depression during COVID-19 pandemic: A global cross-sectional survey. *Progress in Neuro-Psychopharmacology & Biological Psychiatry*, 109, 110236. 10.1016/j.pnpbp.2020.110236 33373680 PMC7834119

[cit0044] Wilson, T. D., Meyers, J., & Gilbert, D. T. (2003). “How happy was I, anyway?” A retrospective impact bias. *Social Cognition*, 21(6), 421–446. 10.1521/soco.21.6.421.28688

[cit0045] Xu, Y., Zhang, R., Zhou, Z., Fan, J., Liang, J., Cai, L., Peng, L., Ren, F., & Lin, W. (2021). Parental psychological distress and attitudes towards COVID-19 vaccination: A cross-sectional survey in Shenzhen, China. *Journal of Affective Disorders*, 292, 552–558. 10.1016/j.jad.2021.06.003 34147967 PMC8179837

